# Supplementary material for: Metabolic Abnormalities Linked to Auditory Pathways in ApoE-Knockout HEI-OC1 Cells: A Transcription-Metabolism Co-Analysis
Source: Biomolecules. 2022 Sep 1;12(9):1217. doi: 10.3390/biom12091217 (PMC9496352; doi:10.3390/biom12091217)
Supplement: Supplementary file 1 [file biomolecules-12-01217-s001.zip › biomolecules-1840078-supplementary.pdf]

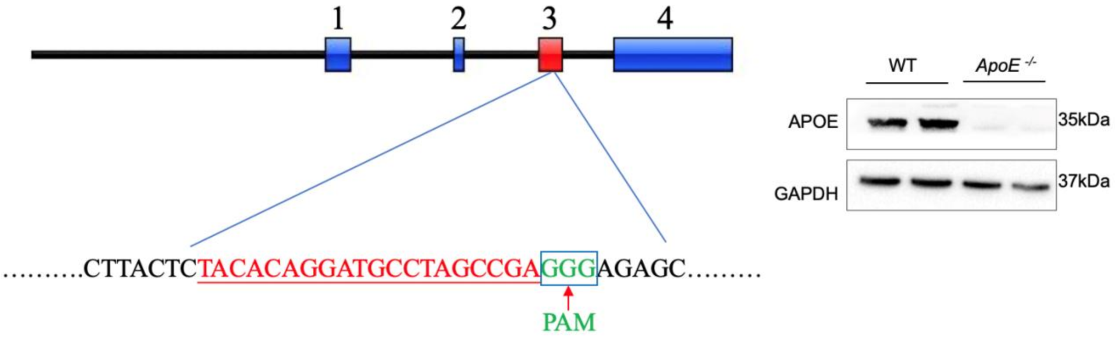

Table S1. Generation of *ApoE*-deficient HEIOC1 cell lines (GAPDH, glyceraldehyde 3-phosphate dehydrogenase, PAM, protospacer adjacent motif).

NO.	Target Site	Genetype
WT	CTTACTCTACACAGGATGCCTAGCCGAGGGAGAGC<br>CTTACTCTACACAGGATGCCTAGCCGAGGGAGAGC	+/+
MUT-1	CTTACTCTACACAGGATGCCTAGC…AGGGAGAGC<br>CTTACTCTACACAGGATGCCTAGC…AGGGAGAGC	c. 54-55 del CG
MUT-2	CTTACTCTACACAGGATGCCTAGC..GAGGGAGAGC<br>CTTACTCTACACAGGATGCCTAGC..GAGGGAGAGC	c. 54 del C

Table S2. The primer sequences of qPCR (F, forward, R, reverse, GAPDH, glyceraldehyde. 3-phosphate dehydrogenase, *Scl7a8*, solute carrier membrane transport protein, family 7, subfamily a, member 8, *Scl6a19*, solute carrier membrane transport protein, family 6, subfamily a, member 19).

**Gene**		**5′-3′**
Mus-qPCR-Gapdh	F	TGGCCTTCCGTGTTCCTAC
	R	GAGTTGCTGTTGAAGTCGCA
Mus-qPCR-Slc7a8	F	TGTGACTGAGGAACTTGTGGA
	R	GTGGACAGCAACAGAAATG
Mus-qPCR-Sl6a19	F	CAGGTGCTCAGGTCTTCTACT
	R	CGATCAGAATCCATCTCACAA

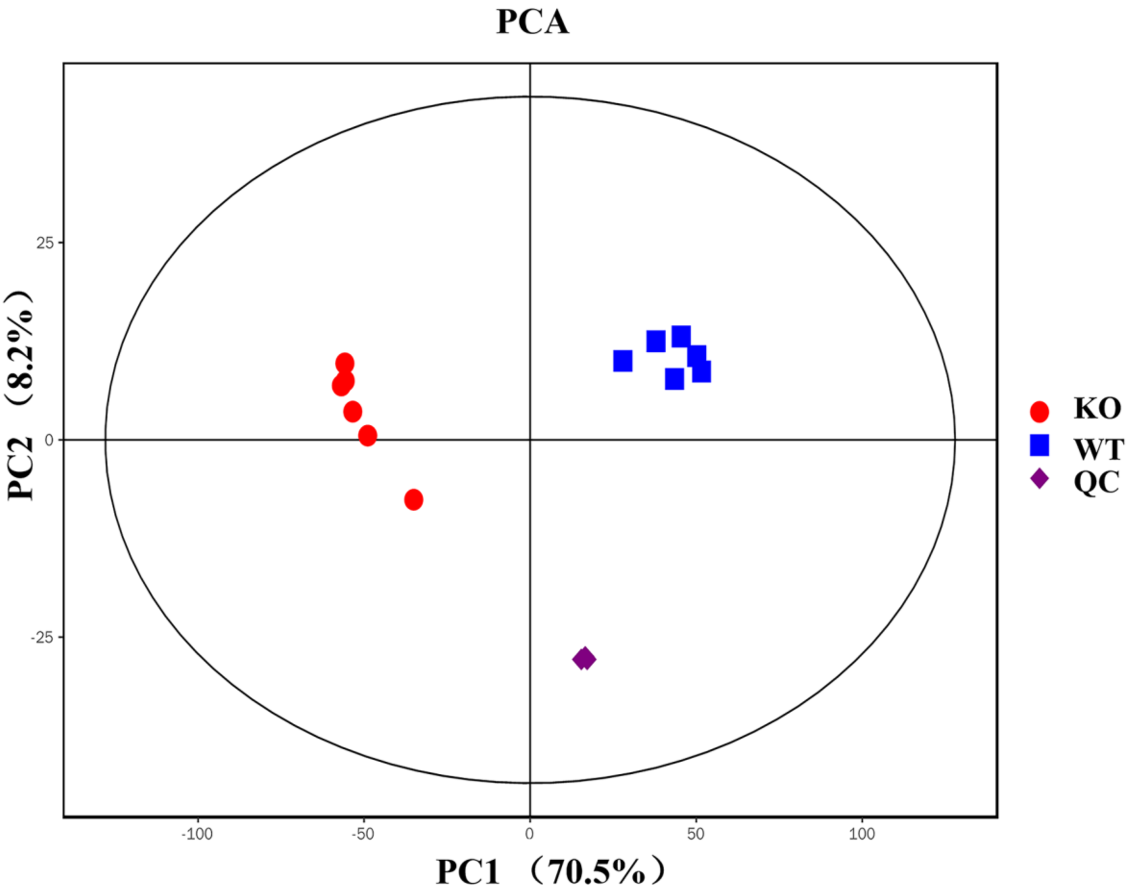

Figure S1. Metabolome data quality control results. PCA analysis of metabolome data (n = 6).

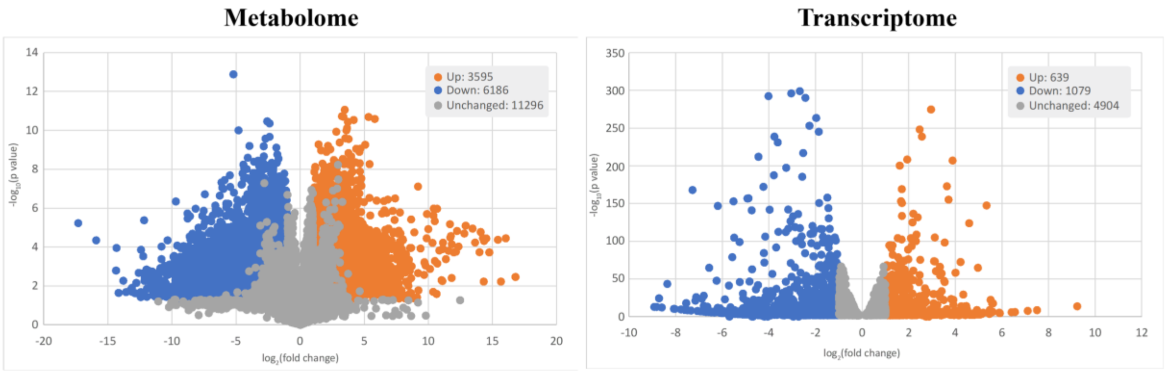

Figure S2. Volcanic map of metabolome and transcriptome differences. Volcanic map of differential metabolites (left) and differentially expressed genes (right). Each origin represents a detected substance, the red mark is upregulated, the blue mark is downregulated, and the gray is the detected substance with no significant difference. The significant difference substance screening criteria are *p* value < 0.05, |log2FC| ≥ 1, VIP > 1.

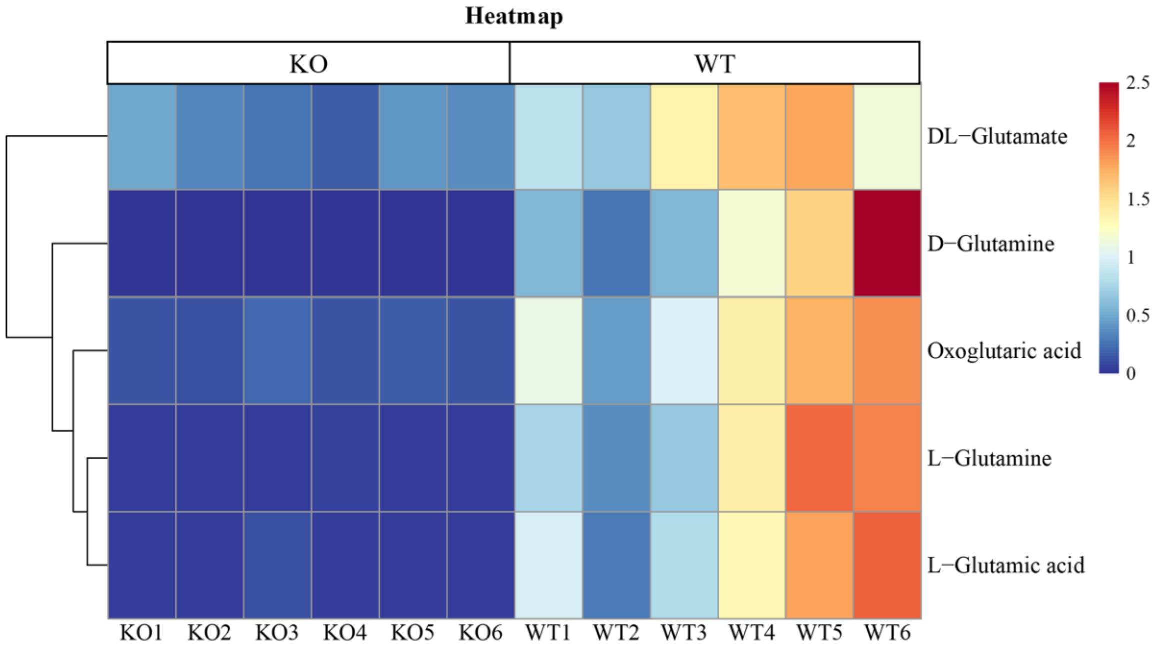

Figure S3. Heatmap of glutamate metabolites.

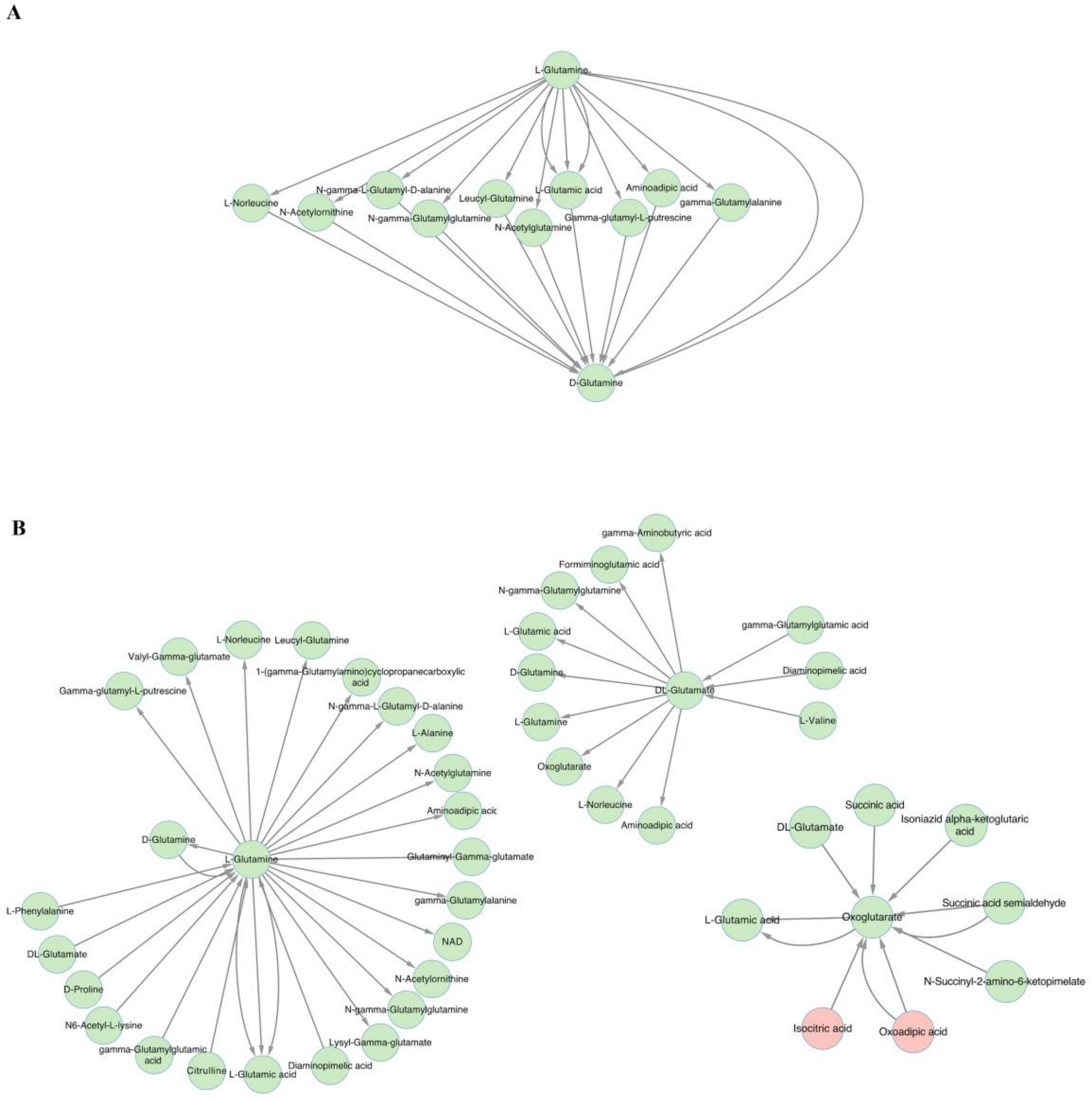

Figure S4. Metabolite association analysis network (NAD, nicotinamide adenine dinucleotide). (A) Intermediate metabolites of the L-glutamine to D-glutamine transformation pathway; (B) Metabolite association network of L-glutamine, DL-glutamate, and oxoglutarate.

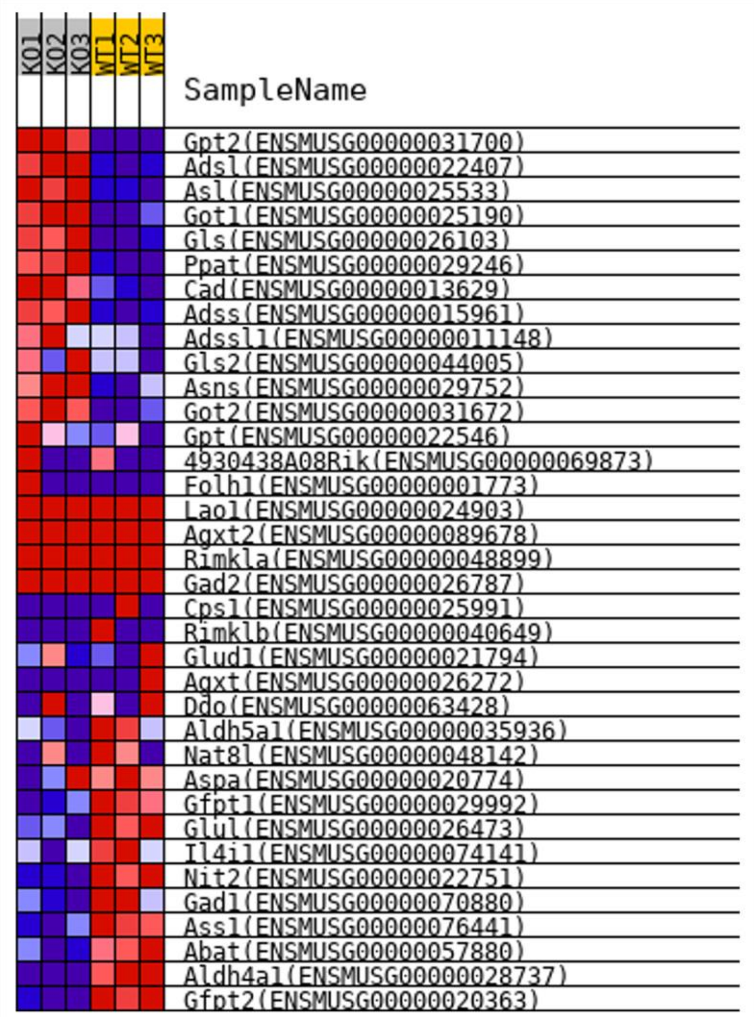

Figure S5. Gene expression in the alanine, aspartate, and glutamate metabolic pathway (*Gpt2*, Glutamine-pyruvate transaminase 2, *Adsl*, adenylosuccinate lyase, *Asl*, argininosuccinate lyase, *Got1*, glutamic-oxaloaceitc transaminase 1, *Gls*, glutaminase, *Ppat*, phosphoribosyl pyrophosphate amidotransferase, *Cad*, carbamoyl-phosphate synthetase 2, *Adss*, adenylosuccinate synthetase, *Adssl1*, adenylosuccinate synthetase like 1, *Gls2*, glutaminase 2, *Asns*, asparagine synthetase, *Got2*, glutamic-oxaloacetic transaminase 2, *Gpt*, glutamic-pyruvic transaminase, *4930438A08Rik*, RIKEN cDNA 4930438A08 gene (Riken Research Institute, Japan), *Folh1*, folate hydrolase 1, *Lao1*, L-amino acid oxidase 1, *Agxt2*, alanine-glyoxylate aminotransferase 2, *Rimkla*, ribosomal modification protein rimK like family member A, *Gad2*, glutamate decarboxylase 2, *Cps1*, carbamoyl-phosphate synthase 1, *Rimklb*, ribosomal modification protein rimK like family member B, *Glud1*, glutamate dehydrogenase 1, *Agxt*, alanine-glyoxylate and serine- pyruvate aminotransferase, *Dod*, D-aspartare oxidase, *Aldh5a1*, aldehyde dehydrogenase 5 family member A1, *Nat8l*, N-acetytransferase 8 like, *Aspa*, aspartoacylase, *Gfpt1*, glutamine-fructose-6-phosphate transaminase 1, *Glul*, glutamate-ammonia ligase, *Il4i1*, interleukin 4 induced 1, *Nit2*, nitrilase family member 2, *Gad1*, glutamate decarboxylase 1, *Ass1*, argininosuccinate synthase 1, *Abat*, 4-aminobutyrate aminotransferase, *Aldh4a1*, aldehyde dehydrogenase 4 family member A1, *Gfpt2*, glutamine-fructose-6-phosphate transaminase 2).

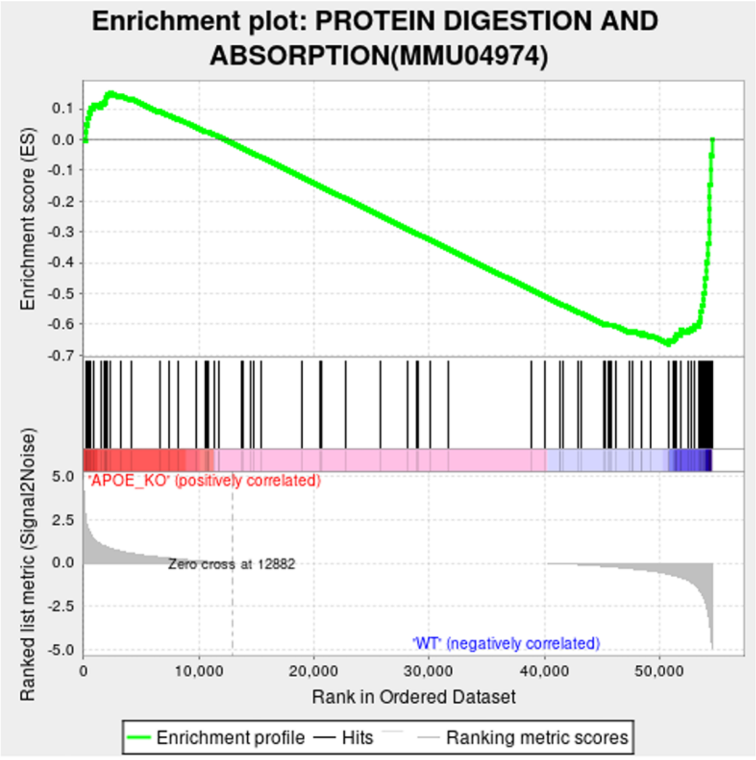

Figure S6. GSEA analysis of protein digestion and absorption. The green curve corresponds to the enrichment fraction of each gene in the pathway, and the enrichment index of genes with upregulated expression is greater than 0, whereas it is less than 0. There is an obvious peak in the negative value of the curve in the picture, which indicates that the pathway is downregulated as a whole.

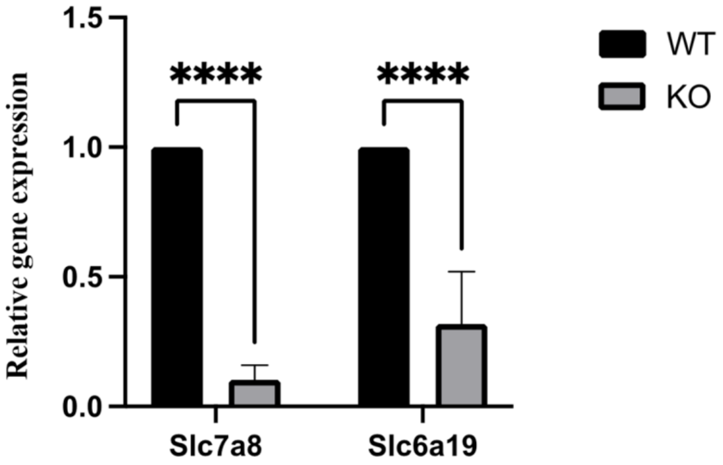

Figure S7. qPCR assay for *Slc7a8* and *Slc6a19* gene expression. **** $p < 0.0001$.
